# Oxytocin-Trust Link in Oxytocin-Sensitive Participants and Those Without Autistic Traits

**DOI:** 10.3389/fnins.2021.659737

**Published:** 2021-05-25

**Authors:** Hirofumi Kurokawa, Yusuke Kinari, Hiroko Okudaira, Kiyotaka Tsubouchi, Yoshimichi Sai, Mitsuru Kikuchi, Haruhiro Higashida, Fumio Ohtake

**Affiliations:** ^1^School of Economics and Management, University of Hyogo, Kobe, Japan; ^2^Hirao School of Management, Konan University, Nishinomiya, Japan; ^3^Doshisha Business School, Doshisha University, Kyoto, Japan; ^4^Department of Hospital Pharmacy, Kanazawa University, Kanazawa, Japan; ^5^Department of Psychiatry and Neurobiology, Graduate School of Medical Science, Kanazawa University, Kanazawa, Japan; ^6^Research Center for Child Mental Development, Kanazawa University, Kanazawa, Japan; ^7^Center for Infectious Disease Education and Research, Osaka University, Suita, Japan

**Keywords:** oxytocin, human, trust, interindividual variation, saliva

## Abstract

There have been numerous studies in which the biological role of oxytocin in trusting behavior has been investigated. However, a link between oxytocin and trust in humans was discovered only in one early study. We hypothesized that there is a large interindividual variation in oxytocin sensitivity, and that such variation is one reason for the doubt surrounding the role of oxytocin in trusting behavior. Here, in a double-blind, prospective, case-control study, we administered intranasal oxytocin to participants of trust and risk games. We measured salivary oxytocin concentration, relating it to the amount of money transferred among participants (a proxy for trust) and the autism-spectrum quotient (AQ). A one-sided Fisher’s exact test was performed to detect differences between the oxytocin and placebo groups in the proportions of investors who transferred the maximum amount of money. We discovered a tendency for participants who received oxytocin to transfer higher amounts of money to co-participants than those who received a placebo (*P* = 0.04). We also revealed a high degree of interindividual variation in salivary oxytocin concentrations after oxytocin administration. After stratifying the samples with respect to oxytocin sensitivity, oxytocin-sensitive participants in the oxytocin group also transferred higher amounts of money than those in the placebo group (*P* = 0.03), while such a tendency was not observed for oxytocin-insensitive participants (*P* = 0.34). Participants with lower AQ scores (less severe autistic traits) exhibited a greater tendency toward trusting behavior after oxytocin administration than did those with higher AQ scores (*P* = 0.02). A two-sample *t*-test that was performed to detect significant differences in the mean transfers between the oxytocin and placebo groups indicated no significant between-group difference in the mean transfers (*P* = 0.08). There are two possible interpretations of these results: First, there is no effect of oxytocin on trust in humans; second, the effects of oxytocin on trust in humans is person-dependent. However, the results should be interpreted with caution as the effect size was not larger than the minimal detectable effect size and the results were not statistically significant (*P* > 0.05) after Bonferroni corrections.

## Introduction

Oxytocin is a neuropeptide that plays a key role in female reproductive function, such as facilitating parturition and milk ejection during lactation. It also promotes social attachment and regulates social behaviors in several mammals, including humans. For example, oxytocin improves an individual’s ability to produce normative ratings of others’ emotions (Domes et al., 2007; Guastella et al., 2010) and increases traits that facilitate interpersonal relations, such as generosity and cooperation (Zak et al., 2007; Declerck et al., 2010). However, Bartz et al. (2011a) reported that the effects of oxytocin on social cognition and prosociality are often weak and/or inconsistent. In a meta-analysis, Leppanen et al. (2017) also discovered no effects of oxytocin on individuals’ score in the “Reading the Mind in the Eyes” test.

Oxytocin is also recognized to play a biological role in trust in humans. Kosfeld et al. (2005) reported on their pioneering study in which intranasal administration of oxytocin and a now-standard trust-game context were used to link oxytocin with trust in humans. In the trust game, participants are randomly assigned the role of investor or trustee. First, the investor decides on an amount of money to transfer to the trustee. The experimenter triples the investment and the trustee receives the tripled amount. Finally, the trustee decides on the amount to return to the investor. The results revealed that intranasal oxytocin increased the amount that investors transferred. An additional experiment on risk attitude revealed that intranasal oxytocin did not affect the subject’s risk attitude, indicating that intranasal oxytocin elicited trust among subjects.

Such a causal link between oxytocin and trust in humans, however, has not been corroborated in other research. Nave et al. (2015) reviewed six studies (Baumgartner et al., 2008; Barraza, 2010; Mikolajczak et al., 2010; Ebert et al., 2013; Klackl et al., 2013; Yao et al., 2014) in which unsuccessful attempts were made to replicate the results of Kosfeld et al. (2005). Although some of the details of the experiments in these studies were modifications of the original, the underlying principles were essentially the same: intranasal administration of oxytocin and a trust game. Declerck et al. (2020) pointed out that those studies were not direct replications in the sense that the investors and trustees did not have any contact with each other. The participants in Kosfeld et al. (2005) were allowed approximately 5 min to talk to each other after administration of oxytocin or a placebo. Declerck et al. (2020) emphasized the importance of this “Minimal Social Contact” in conducting their own replication study. However, their results also did not provide evidence for an oxytocin-trust link, although they confirmed that participants with a high disposition to trust tended to transfer maximal amounts of money in the trust game.

Recent studies raised doubt in terms of not only the possible link between oxytocin and trust in humans but also the whole field of research employing intranasal oxytocin and measuring psychosocial outcomes. Walum et al. (2016) demonstrated that intranasal oxytocin studies are considerably underpowered and there is a high probability that most of the published findings do not represent true effects. Lane et al. (2016) suggested the possibility of a “file drawer” problem, whereby negative findings may be hiding in many laboratories’ file drawers. According to that line of reasoning, the effects of intranasal oxytocin may not truly exist, but are artificial. Leng and Ludwig (2016) reported that large concentrations of intranasal oxytocin are required to induce detectable behavioral effects, and questioned whether such could even be attained via the intranasal route. Mierop et al. (2020) summarized these concerns as statistical and methodological problems. Further analysis is needed to understand the true effects of oxytocin on human behavior, and to move the intranasal oxytocin field forward. As highlighted by the cited studies, such research has to be transparent and null results should be reported.

Various studies have suggested that the effect of oxytocin is likely to be person- and/or situation-dependent. Bendix et al. (2015) discovered that plasma oxytocin levels varied across participants and was correlated to personality traits such as monotony avoidance and impulsiveness. In addition, previous studies have indicated that the effect of oxytocin depends on personal conditions such as borderline personality disorder and attachment during childhood (Bartz et al., 2010b, 2011b). The positive effects of oxytocin on sociality depend on receptor genotype (Marsh et al., 2012; Kosaka et al., 2016). Oxytocin sensitivity and autistic traits could be candidates to elicit such person-dependent oxytocin effects.

In this study, we investigated the above positive causal link using the same experimental framework, but modifying it in one main way: measuring participants’ salivary concentrations of oxytocin. We hypothesized that oxytocin sensitivity varies greatly across individuals and that performing subgroup analyses of oxytocin-sensitive participants would provide evidence for a causal link between oxytocin and trust. In many studies, indices, such as the empathy quotient (EQ), autism-spectrum quotient (AQ), and systemizing quotient (SQ), have been used as proxies for autism (Dawson et al., 2002; Hoekstra et al., 2007; Valla et al., 2010), and oxytocin-induced prosocial behavior is correlated to these indices (Bartz et al., 2010a, 2019; Hirosawa et al., 2012; Yamasue et al., 2020). Therefore, we also analyzed trust behavior in relation to autistic traits.

## Materials and Methods

### Participants

We conducted the experiment on October 4, 11, 13, and 25, as well as on November 1, 2017. We recruited 192 participants from Osaka University, where the experiment took place; all were healthy, non-smoking, male students. They were instructed in advance not to take food or drink, other than water, for 2 h before the experiment, and to abstain from alcohol and caffeine starting the evening preceding the experiment. They were also informed that they would be participating in an experiment involving intranasal administration of oxytocin. All participants provided written, informed consent for inclusion in the study, and the experimental protocol was pre-approved by the Osaka University Ethics Committee. All methods were performed in accordance with the relevant guidelines and regulations. We surveyed participants’ AQ, EQ, and SQ scores before the start of the experiments. Most participants answered the survey on the day before the experiment, while a few answered the survey immediately before the experiment started.

### Drug Protocol

Oxytocin (40 international units [IU]/mL; Syntocinon Spray, Sigma-Tau Industrie Farmaceutiche, Riunite S.p.A., Rome, Italy) was prepared by the clinical pharmacy at the Kanazawa University Hospital. The placebo (containing chlorobutanol, methyl p-hydroxybenzoate, propyl p-hydroxybenzoate, and anhydrous citric acid) was prepared by the same pharmacy. The placebo and oxytocin spray containers were prepared to look identical, and the pharmacy randomly assigned them to participants via counterbalancing. Both researchers and participants were blinded to the content of the spray. The participants received a dose of 24 IU (48 μg) oxytocin (three puffs per nostril), in accordance with most studies on intranasal oxytocin in adults (MacDonald and Feifel, 2014).

### Oxytocin Administration

We conducted a double-blind study to compare the trust behavior between experimental groups. Participants were randomly assigned to the oxytocin or placebo group; there were 96 participants per group. Participants who had neither eaten nor drunk for at least 2 h prior to the experiment were seated for 20 min and received instruction on the experiment as well as on self-administration of the spray (Supplementary Figure 1). While inhaling the puff of spray in one nostril, the other nostril was closed by applying pressure to it with the index finger. To reduce the loss of oxytocin or placebo from the nostril, participants tilted their head back for 30 s. Participants were instructed to drink only minimal amounts of water during the experiment, and to wait together in the rest area for approximately 10 min after administration of the spray. The participants sat at a round table and were permitted to converse, satisfying the Minimal Social Contact condition emphasized by Declerck et al. (2020).

### Collection of Saliva Samples and Oxytocin Measurement

The saliva samples were collected before oxytocin administration and after the completion of our experiment, approximately 72 min after administration. We also collected the saliva samples immediately before the trust game (but after oxytocin administration and interaction with other participants). For each collection, participants washed their mouth twice with bottled water. After approximately 5 min, they held the opening of a sterile, 15-mL polypropylene tube (Greiner Bio-one Co. Ltd., Tokyo, Japan) in their mouth and let their saliva (0.5–1.5 mL) flow directly into the tube for approximately 2 min. These tubes were immediately placed on ice and subsequently frozen at −20°C. The next day, all the samples were shipped on dry ice from Osaka to Kanazawa, where it was stored at −80°C upon arrival. Two to seven days later, the samples were thawed. The precipitates were removed after two centrifugations at 4°C at 1,500 × g for 10 min each. Aliquots of 100 μL were stored in 1.5-mL microtubes at −80°C until oxytocin measurement. The oxytocin concentrations of these samples were measured in 96-well plates with oxytocin antibodies (Enzyme immunoassay kit, Enzo Life Sciences, Inc., Farmingdale, NY, United States), as described previously (Yuhi et al., 2018; Tanaka et al., 2020). The saliva samples were not treated with solid-phase extraction columns. The optical density of the samples and standards was measured at a wavelength of 405 nm using a microplate reader (Bio-Rad Laboratories, Hercules, CA, United States). Measurements were performed in duplicate. Sample concentrations were calculated according to the relevant standard curve. The intra- and interassay coefficients were both <12%.

Unextracted human saliva samples have been used for oxytocin measurements (Carter et al., 2007; White-Traut et al., 2009). This method was validated for use with dog saliva (MacLean et al., 2017). However, certain salivary components have been discovered to interact with labeled oxytocin antibodies (McCullough et al., 2013; Leng and Sabatier, 2016; MacLean et al., 2017). To examine the possibility of such interactions in our study, we measured saliva samples spiked with oxytocin (0–250 pg/mL). The enzyme immunoassay values obtained were proportional to the spiked oxytocin concentrations, similar to the discovery made by Yuhi et al. (2018), suggesting that the monitored values were reliable in determining the difference between oxytocin concentrations before and after the experiment, as well as the ratio of the two.

### The Trust Game

Upon returning to the laboratory, we conducted the trust game, in which pairs of participants interacted as trustees and investors; participants were blinded in terms of the pairings. All participants’ decisions were entered into a computer using z-Tree software (Fischbacher, 2007). The settings for the trust game were essentially the same as those of Kosfeld et al. (2005). Participants received 12 monetary units (MU) as an initial endowment and were randomly assigned to the role of investor or trustee. The investor could transfer 0, 4, 8, or 12 MU to the trustee, which the experimenter subsequently tripled. The trustee was informed of the amount that the investor had invested and decided on the amount to send back to the investor, which could be any amount from 0 MU to his total holdings. The remainder would be retained by the trustee. According to the trustee’s decision, the MU amounts were exchanged for real money based on a pre-announced rate of 1 MU = 40 yen. The trust game was performed four times. The roles assigned to the participants were the same for all four rounds, but pairs of participants were randomly assigned per round.

### The Risk Game (Control)

We also conducted a risk game according to the methods of Kosfeld et al. (2005). This game served to control for possible effects of oxytocin on risk-taking behavior. While participants were randomly assigned to the role of investor or trustee in the trust game, all participants played the role of investor in the risk game. The role of trustee was performed by a computer program that randomly selected the amount returned to the investor based on a specific distribution derived from the literature (Johnson and Mislin, 2011). The risk game was also performed four times. In the trust game, the amount of MU that the investor transfers depends on his response to the risk arising from uncertainty of the trustee’s behavior as well as his level of trust in the trustee. In the risk game, however, the investor does not need to consider the trust issue. Therefore, if there is a group difference in behavior in the trust game but not in the risk game, the difference is associated with trust. On the other hand, if we find a group difference in behavior in both the trust and risk games, the difference is not only due to trust.

### Relation to AQ, EQ, and SQ Scores

To analyze the effect of oxytocin on trusting behavior in relation to autistic traits, we selected the AQ, EQ, and SQ scores as proxies for autism. These indices have been used as proxies for autism in many studies (Dawson et al., 2002; Hoekstra et al., 2007; Valla et al., 2010), and oxytocin-induced prosocial behavior is correlated to these indices (Bartz et al., 2010a, 2019; Hirosawa et al., 2012; Yamasue et al., 2020). AQ test is developed by Baron-Cohen et al. (2001) and the score of AQ test is used as a screening measure of the degree to which an adult with normal intelligence has autistic traits. For example, a score of 25 or less indicates that a diagnosis of Asperger’s syndrome can effectively be ruled out (Woodbury-Smith et al., 2005). Following the study by Woodbury-Smith et al. (2005), we divided participants into two groups: those with an AQ score higher than 25 (likely to be diagnosed with high-functioning autism) and those with an AQ score of 25 or less (unlikely to be diagnosed with high-functioning autism). Dividing the sample based on the median AQ score, 21, yielded essentially the same results. We also divided participants based on the median EQ and SQ scores (16 and 18, respectively).

### Statistical Analysis

The significance level was set to 5% for all statistical tests performed in this study. A one-sided, two-sample *t*-test assuming equal variance was performed to detect significant differences in the mean transfer of MU and the initial oxytocin concentration between groups, and a one-sided Student’s *t*-test was performed for pairwise comparisons. A one-sided, paired *t*-test was performed to detect significant differences in the mean oxytocin concentration at three time points within groups. A one-sided Fisher’s exact test was performed to detect differences between the oxytocin and placebo groups in the proportions of investors who transferred the maximum MU. A two-sample Kolmogorov-Smirnov test for equality of distribution functions was performed to detect differences in the distributions of salivary oxytocin concentrations between the oxytocin and placebo groups.

## Results

### MU Transfers Following Oxytocin or Placebo

**FIGURE 1:**
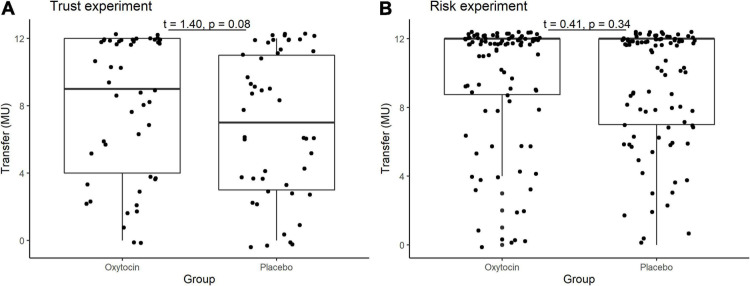
Box plots of average transfer per investor in the trust **(A)** and risk **(B)** games. For each plot, the middle horizontal line denotes the median value; the box extends from the 25th to the 75th percentile values; the vertical lines extending from the box denote adjacent values (i.e., the most extreme values within 1.5 interquartile of the 25th and 75th percentile of each group); and the dots denote the average transfer per investor.

In the trust game, we observed essentially the same tendency as Kosfeld et al. (2005). Table 1 contains the mean and median transfers averaged over four rounds of trust or risk gaming. Figure 1 contains box plots of the average transfer per investor. In the trust game, there was no statistically significant difference in investment between participants who received oxytocin and those who received a placebo. In the trust game, the difference in mean average transfer in the oxytocin group was not significantly higher than that in the placebo group [8.1 vs. 6.9; *t*_(__94__)_ = 1.40, *P* = 0.08, one-sided]. The median average transfer in the oxytocin group was larger than that in the placebo group (9 and 7, respectively). In the risk game, the difference in mean average transfers between groups was not statistically significant [9.6 vs. 9.4; *t*_(__190__)_ = 0.41, *P* = 0.34, one-sided].

**TABLE 1:** Transfer behavior of investors.

	**Trust experiment**			**Risk experiment**		
	**Oxytocin group**	**Placebo group**	**Difference**	**Oxytocin group**	**Placebo group**	**Difference**
Mean average transfer (MU)	8.1	6.9	1.2 (117.5%)	9.6	9.4	0.2 (102.2%)
Median average transfer (MU)	9	7	2.0 (128.6%)	12	12	0.0 (100.0%)
Standard deviation of transfers (MU)	4.2	4.3	−0.1 (97.5%)	3.7	3.4	0.3 (109.3%)
Number of observations	48	48		96	96	

*The mean and median transfer of monetary units (MU), averaged over four rounds in the trust and risk experiments, indicated that, in the trust experiment, participants who received oxytocin transferred more MU than did participants who received a placebo. The number of observations were 48 and 96 in the trust and risk experiments, respectively, as, in the former, half of the participants were assigned the role of investor, while, in the latter, all participants played the role of investor. The mean transfer in the oxytocin group was not significantly higher than that in the placebo group [8.1 vs. 6.9; t*_(_*_94__)_ = 1.40, P = 0.08, one-sided]. The median transfer in the oxytocin group was greater than that in the placebo group (9 vs. 7). In the risk experiment, the intergroup differences in mean transfer was also not statistically significant [t*_(_*_190__)_ = 0.41, P = 0.34, one-sided]. The figures in parentheses describe an increase in the oxytocin group relative to that in the placebo group.*

**FIGURE 2:**
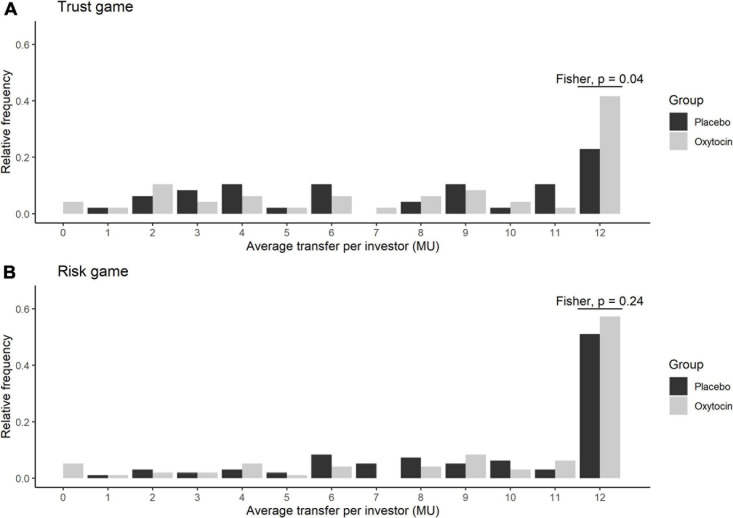
Relative frequency of the average transfers in the oxytocin and placebo groups. The x axis represents the average transfer per investor. For instance, if the average transfer per investor equals 12, it means that the investor sent the maximum amount of MU in all four rounds. The y axis represents the relative frequencies of investors for each average transfer. Black and white bars represent the relative frequency of the average transfers in the placebo and oxytocin groups, respectively. Participants who received oxytocin demonstrated a greater degree of trust than those who received placebo. In the trust experiment **(A)**, 20 of 48 participants in the oxytocin group transferred the maximum amount (12 MU), whereas only 11 of 48 participants in the placebo group did the same (Fisher’s exact test; *P* = 0.04; one-sided). In the risk experiment **(B)**, we did not observe a significant difference in this behavior between the groups (Fisher’s exact test; *P* = 0.24; one-sided).

Figure 2 illustrates the relative frequencies of investors’ average transfers over four rounds of the trust game (**A**) and the risk game (**B**) in the oxytocin (white bars) and placebo (black bars) groups. The average transfer amount in the trust game was 12 MU for 20 out of 48 participants (42%) in the oxytocin group, whereas it was 12 MU for only 11 out of 48 participants (23%) in the placebo group. This intergroup difference, i.e., in those who exhibited the maximum level of trust, was significant in the trust game (Fisher’s exact test; *P* = 0.04, one-sided), but not in the risk game (Fisher’s exact test; *P* = 0.24, one-sided).

Both oxytocin and placebo groups transferred more MU in the risk game (overall mean, 9.5) than in the trust game (mean, 7.5), indicating that participants in our experiment tended to trust a randomization algorithm more than they trusted fellow participants. Restricting the sample to the 96 participants who were investors in the trust game, the increase in transfer from the trust game to the risk game for the oxytocin group was significantly smaller than that for the placebo group [0.98 vs. 2.79; *t*_(__94__)_ = 1.75, *P* = 0.04]. This may be interpreted as the effect of oxytocin in increasing trust in fellow participants. That is, oxytocin elicited trust in fellow humans to a level nearly equaling that of a randomization algorithm.

**FIGURE 3:**
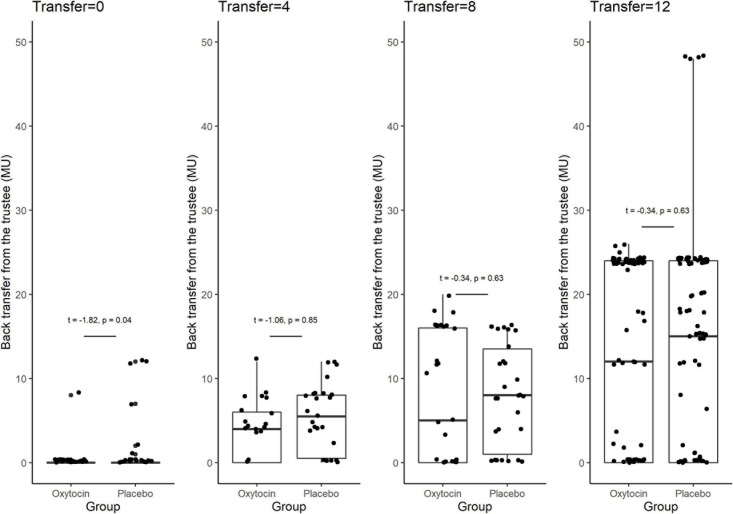
Box plots of back transfer. The back transfer for different invested amounts is indicated separately for the oxytocin and placebo groups. For each plot, the middle horizontal line denotes the median value; the box extends from the 25th to the 75th percentile values; the vertical lines extending from the box denote adjacent values (i.e., the most extreme values within 1.5 interquartile of the 25th and 75th percentile of each group); and the dots denote back transfers. For the different non-zero invested amounts, we observed no significant difference in the mean back transfer between trustees in the oxytocin and placebo groups [transfer = 4, 4.090 vs. 5.269, *t*_(__46__)_ = −1.06, *P* = 0.85, one-sided; transfer = 8, 7.355 vs. 7.967, *t*_(__59__)_ = −0.34, *P* = 0.63, one-sided; transfer = 12, 12.516 vs. 13.258, *t*_(__182__)_ = −0.42, *P* = 0.66, one-sided]. For transfers of 0 MU, the participants in the oxytocin group returned less MU than those in the placebo group [0.167 vs. 1.070, *t*_(__89__)_ = −1.82, *P* = 0.04, one-sided]. We observed no significant difference in the average back transfer between trustees in the oxytocin and placebo groups [7.630 vs. 8.620; *t*_(__94__)_ = 0.71, *P* = 0.24].

Figure 3 provides box plots of the back transfer for different invested amounts for the oxytocin and placebo groups, revealing that oxytocin did not affect participants’ reciprocity. For the different non-zero invested amounts, we observed no significant difference in the mean back transfer between trustees in the oxytocin and placebo groups [transfer = 4, 4.090 vs. 5.269, *t*_(__46__)_ = −1.06, *P* = 0.85, one-sided; transfer = 8, 7.355 vs. 7.967, *t*_(__59__)_ = −0.34, *P* = 0.63, one-sided; transfer = 12, 12.516 vs. 13.258, *t*_(__182__)_ = −0.42, *P* = 0.66, one-sided]. For transfers of 0 MU, the participants in the placebo groups returned more MU than those in the oxytocin group [0.167 vs. 1.070, *t*_(__89__)_ = −1.82, *P* = 0.04, one-sided], indicating that oxytocin does not enhance participants’ reciprocity. There was no significant difference in the average back transfer between the oxytocin and placebo groups [7.630 vs. 8.620; *t*_(__94__)_ = 0.71, *P* = 0.24]. These results are consistent with that of Kosfeld et al. (2005), that oxytocin does not increase the general inclination to behave prosocially.

### Variance in Sensitivity to Oxytocin Administration

**FIGURE 4:**
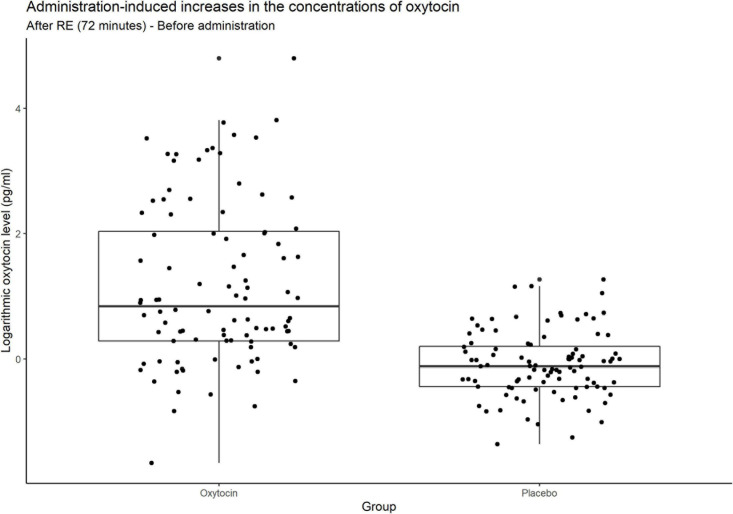
Box plots of administration-induced increases in the concentrations of oxytocin. For each plot, the middle horizontal line denotes the median value; the box extends from the 25th to the 75th percentile values; the vertical lines extending from the box denote adjacent values (i.e., the most extreme values within 1.5 interquartile of the 25th and 75th percentile of each group); and the dots denote increases in the concentrations of oxytocin per investor from before administration to after the risk experiment. In the oxytocin group, the concentration of oxytocin surged in some participants, whereas there were small and even negligible increases in others. As expected, many participants in the placebo group exhibited almost no change in the concentration of oxytocin.

Figure 4 contains box plots of administration-induced increases in the concentrations of oxytocin for the oxytocin and placebo groups. In that group, the concentration of oxytocin surged in some participants, whereas relatively small or negligible increases were elicited in others. In other words, the oxytocin group contained a mixture of oxytocin-sensitive and oxytocin-insensitive participants.

**FIGURE 5:**
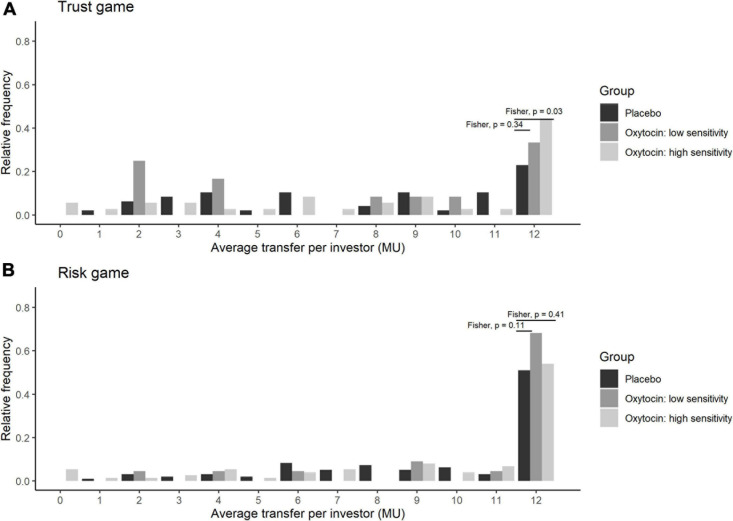
Transfer amounts in the trust **(A)** and risk **(B)** games, stratified by sensitivity to oxytocin. The bars indicate the relative frequencies of the different invested amounts in monetary units (MU) averaged for all four rounds. White, gray, and black bars represent the relative frequencies in the high-sensitivity, low-sensitivity, and placebo groups, respectively. High and low sensitivity were defined as post-administration increases in oxytocin concentration being above and below the median increase, respectively. The x-axis represents the average transfer per investor. For instance, if the average transfer per investor equals 12, it means that the investor sent the maximum amount of MU in all four rounds. The difference in the number of participants who invested the maximum amount between the high-sensitivity (*n* = 36) and placebo (*n* = 48) groups was significant (Fisher exact test; *P* = 0.03, one-sided). In contrast, the corresponding difference between the placebo and low-sensitivity (*n* = 12) groups was not significant (Fisher exact test; *P* = 0.34, one-sided).

**FIGURE 6:**
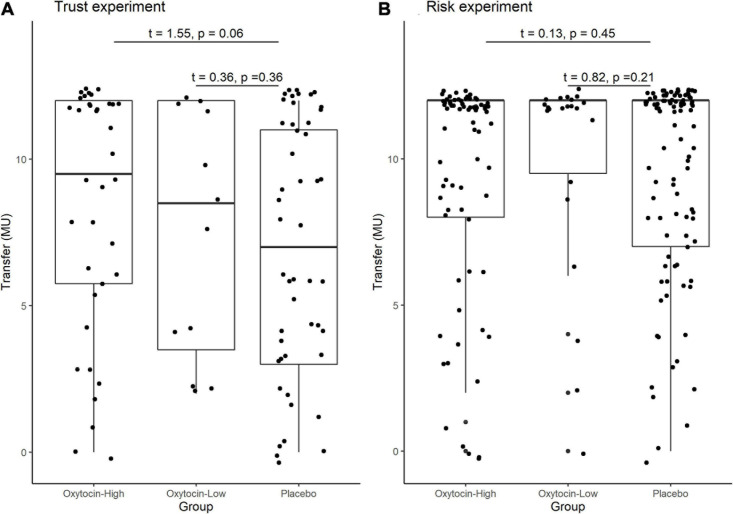
Box plots of average transfer per investor in the trust **(A)** and risk **(B)** games, stratified by sensitivity to oxytocin. For each plot, the middle horizontal line denotes the median value; the box extends from the 25th to the 75th percentile values; the vertical lines extending from the box denote adjacent values (i.e., the most extreme values within 1.5 interquartile of the 25th and 75th percentile of each group); and the dots denote the average transfer per investor. The difference in average investment in the trust game was significant neither between the high-sensitivity and placebo groups [8.361 vs. 6.917; *t*_(__82__)_ = 1.55, *P* = 0.06, one-sided], nor between the low-sensitivity and placebo groups [7.417 vs. 6.917; *t*-test; *t*_(__58__)_ = 0.36, *P* = 0.36, one-sided].

Stratifying the oxytocin group with respect to the sensitivity to oxytocin provided clearer evidence. Figure 5 illustrates the relative frequency of different invested amounts averaged over all four rounds in the trust and risk games [panels (**A**) and (**B**), respectively] stratified according to the high-sensitivity (white bars), low-sensitivity (gray bars), and placebo (black bars) groups. High or low sensitivity to oxytocin was defined as an increase in the oxytocin level above or below the median concentration, respectively. The difference in the number of participants who invested the maximum amount between the high-sensitivity (*n* = 36) and placebo (*n* = 48) groups was significant (Fisher’s exact test; *P* = 0.03, one-sided). In contrast, the difference between the placebo and low-sensitivity groups (*n* = 12) was not significant (Fisher’s exact test; *P* = 0.34, one-sided). Figure 6 contains box plots of average transfer per investor with respect to their sensitivity to oxytocin. The difference in average investment in the trust game was significant neither between the high-sensitivity and placebo groups [8.361 vs. 6.917; *t*_(__82__)_ = 1.55, *P* = 0.06, one-sided], nor between the low-sensitivity and placebo groups [7.417 vs. 6.917; *t*-test; *t*_(__58__)_ = 0.36, *P* = 0.36, one-sided].

### Variance in Baseline Oxytocin Levels

**FIGURE 7:**
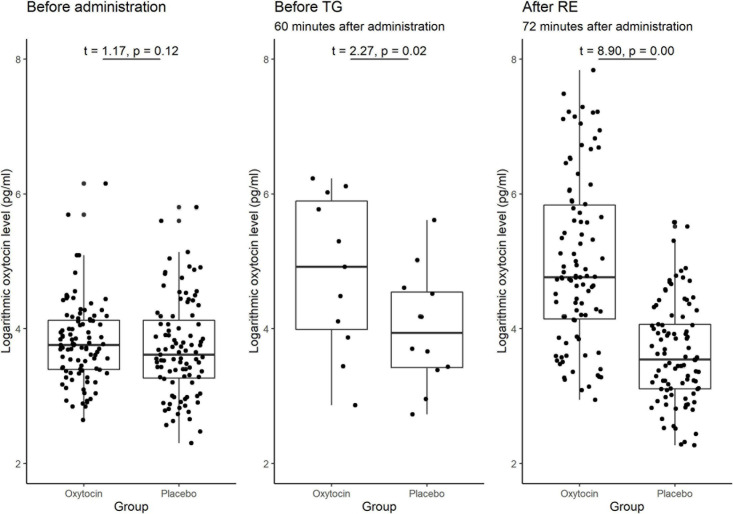
Box plots of oxytocin concentrations at different time points in the oxytocin and placebo groups. For each plot, the middle horizontal line denotes the median value; the box extends from the 25th to the 75th percentile values; the vertical lines extending from the box denotes adjacent values (i.e., the most extreme values within 1.5 interquartile of the 25th and 75th percentile of each group); and the dots denote the oxytocin concentration per investor. The sample size for the time point before the trust game (TG) was small (*n* = 12), as we measured this concentration only for the last experimental session. The oxytocin concentration in the oxytocin group increased immediately after administration and remained elevated until the end of the experiment. The difference in the average oxytocin concentration before administration vs. after the risk experiment (RE) was significant for the oxytocin group [*t*_(__95__)_ = 8.97, *P* < 0.01, one-sided], but not for the placebo group [*t*_(__95__)_ = −1.53, *P* = 0.94, one-sided]. The differences in the oxytocin concentrations before the TG vs. after the RE were not significant in either the oxytocin group [*t*_(__11__)_ = −2.41 *P* = 0.98, one-sided] or the placebo group [*t*_(__11__)_ = −1.22, *P* = 0.88, one-sided]. The difference in the oxytocin concentrations before administration vs. before the TG was significant in the oxytocin group [*t*_(__11__)_ = 2.55, *P* = 0.01, one-sided], but not in the placebo group [*t*_(__11__)_ = 1.38, *P* = 0.10, one-sided].

A substantial difference in the initial (baseline) concentration of oxytocin between the oxytocin and placebo groups may also be the reason for the previous lack of clarity of the possible causal link between oxytocin and trusting behavior. Figure 7 illustrates the participants’ oxytocin concentration for each group before administration, between administration and the start of the trust game, and after the risk game. The difference in the mean initial level of oxytocin between these groups was not significant [*t*_(__190__)_ = 1.17, *P* = 0.12, one-sided]. We also detected no significant difference in the distribution of the initial levels of oxytocin between groups (Kolmogorov-Smirnov test; *P* = 0.15, one-sided). These results suggest that the presence of substantial variation in sensitivity to oxytocin, as opposed to variation in the initial concentration of oxytocin, is a more plausible reason for the weak evidence of a causal link between oxytocin and trusting behavior among participants.

### Oxytocin Time Course

As oxytocin was administered before the trust game, and not again before the risk game, the observed association between oxytocin and trust rather than risk could potentially be due to a decline in oxytocin concentration over time. We verified this hypothesis by measuring salivary oxytocin concentration at different time points. Figure 7 demonstrates the significant difference in the average oxytocin concentration before administration vs. after the risk game was significant for the oxytocin group [*t*_(__95__)_ = 8.97, *P* < 0.01, one-sided] but not for the placebo group [*t*_(__95__)_ = −1.53, *P* = 0.94, one-sided]. Moreover, the difference in oxytocin concentration before the trust game vs. after the risk game was not significant in either the oxytocin group [*t*_(__11__)_ = −2.41, *P* = 0.98, one-sided] or the placebo group [*t*-test; *t*_(__11__)_ = −1.22, *P* = 0.88, one-sided]. These results indicate that the interactions between the investor and the trustee in the trust game do not increase the level of oxytocin. This is further support for a causal link that oxytocin increases trust in humans.

### Oxytocin and Trust Behavior in Relation to the AQ Score

Via our survey, we discovered that there was no significant difference in the average AQ scores between the oxytocin and placebo groups [20.20 vs. 20.24; *t*_(__190__)_ = 0.04, *P* = 0.48]. Table 2 summarizes the transfer behavior in the trust and risk experiments for participants in the oxytocin and placebo groups, stratified by AQ score. Participants with a lower AQ score in the oxytocin group transferred higher amounts than those in the placebo group [*t*_(__77__)_ = 1.77, *P* = 0.04, one-sided], while there was no such difference for participants with a higher AQ score [*t*_(__15__)_ = 0.72, *P* = 0.24, one-sided]. Additionally, in the oxytocin group, the number of participants who transferred the maximum amount was significantly higher for those with a lower AQ score than for those with a higher AQ score (Fisher’s exact test; *P* = 0.02, one-sided); there was no such difference in the placebo (Fisher’s exact test; *P* = 0.44, one-sided). Moreover, in the oxytocin group, the difference in the average transferred amount between lower- and higher-AQ subgroups was 3.3 MU [*t*_(__46__)_ = 2.01, *P* = 0.03, one-sided]. In the placebo group, the corresponding difference was only 0.1 MU [*t*_(__46__)_ = 0.10, *P* = 0.46, one-sided]. In addition, no significant AQ-related differences between groups were observed in the risk experiment. In contrast to the AQ score, we did not discover EQ- and SQ-related differences in the amount of transfers between groups. There were no significant differences in the average EQ and SQ scores between the oxytocin and placebo groups [EQ, 16.65 vs. 16.63, *t*_(__1__88__)_ = 0.02, *P* = 0.49, one-sided; SQ, 19.05 vs. 19.34, *t*_(__187__)_ = 0.20, *P* = 0.42, one-sided]. There were also no significant differences between the oxytocin and placebo groups in the amount transferred by participants with a lower EQ score [*t*_(__39__)_ = 0.83, *P* = 0.20, one-sided] or by those with a higher EQ score [*t*_(__53__)_ = 1.13, *P* = 0.13, one-sided]. In addition, we observed no significant difference between the oxytocin and placebo groups in transfer by participants with a lower SQ score [*t*_(__42__)_ = 0.44, *P* = 0.33, one-sided] or by those with a higher SQ score [*t*_(__50__)_ = 1.50, *P* = 0.07, one-sided].

**TABLE 2:** Transfer behavior of investors, stratified by autism quotient (AQ) score.

**AQ < 26**	**Trust experiment**			**Risk experiment**		
	**Oxytocin group**	**Placebo group**	**Difference**	**Oxytocin group**	**Placebo group**	**Difference**
Mean average transfer (MU)	8.6	6.9	1.7 (124.6%)	9.8	9.3	0.5 (105.3%)
Median average transfer (MU)	10	7	3.0 (142.9%)	12	11	1.0 (109.0%)
Standard deviation of transfer (MU)	4.1	4.2	−0.1 (97.6%)	3.5	3.3	0.2 (106.0%)
Number of observations	41	38		78	76	

**AQ ≥ 26**	**Trust experiment**			**Risk experiment**		
	**Oxytocin group**	**Placebo group**	**Difference**	**Oxytocin group**	**Placebo group**	**Difference**

Mean average transfer (MU)	5.3	6.8	−1.5 (77.9%)	8.8	9.6	−0.8 (91.7%)
Median average transfer (MU)	5	7.5	−2.5 (66.7%)	12	12	0.0 (100.0%)
Standard deviation of transfer (MU)	3.5	4.7	−1.2 (74.5%)	4.4	3.7	0.7 (118.9%)
Number of observations	7	10		18	20	

*The mean, median, and standard deviation monetary-unit (MU) transfers by investors, averaged over four rounds each of the trust and risk experiments, were stratified according to the AQ score. For participants with a lower AQ score, those in the oxytocin group transferred higher amounts than did those in the placebo group [t*_(_*_77__)_ = 1.77, P = 0.04, one-sided], while there was no such tendency for participants with a higher AQ score [t*_(_*_15__)_ = 0.72, P = 0.24, one-sided]. In the oxytocin group, the difference in the mean amount transferred between participants with a lower and higher AQ score was 3.3 MU [t*_(_*_46_*_)_ = 2.01, P = 0.03, one-sided]; in the placebo group, this difference was only 0.1 MU [t-test; t_(_*_46__)_ = 0.10, P = 0.46, one-sided]. In addition, no significant differences were discovered in the risk experiment.*

**FIGURE 8:**
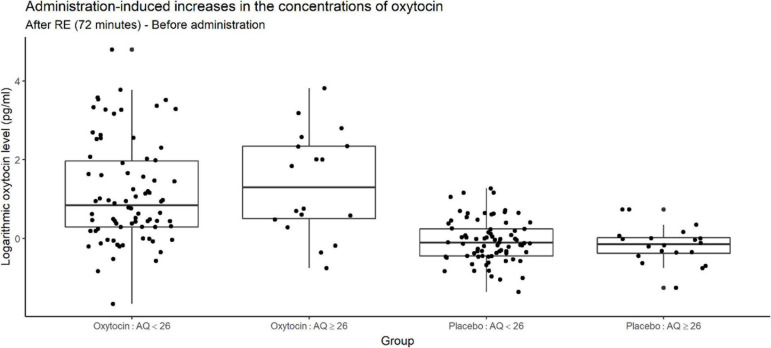
Box plots of administration-induced increases in oxytocin concentration, stratified according to autism quotient (AQ) score. For each plot, the middle horizontal line denotes the median value; the box extends from the 25th to the 75th percentile values; the vertical lines extending from the box denote adjacent values (i.e., the most extreme values within 1.5 interquartile of the 25th and 75th percentile of each group); and the dots denote oxytocin concentration per investor. The figure reveals substantial variation among participants receiving oxytocin in both the higher- and lower-AQ subgroups. The change in salivary oxytocin concentration was measured as the difference between two time points: before administration of oxytocin and after the risk experiment. Certain participants exhibited large increases in the oxytocin concentration, whereas others exhibited very small increases.

Figure 8 depicts increases in the oxytocin concentration upon the administration of oxytocin or placebo, and reveals substantial variation among participants receiving oxytocin in both higher- and lower-AQ groups. Certain participants exhibited large increases in the oxytocin concentration, whereas others exhibited very small increases.

## Discussion

There have been numerous studies in which the biological role of oxytocin in trusting behavior has been investigated. However, their evidence raises doubts regarding a link between oxytocin and trust among humans. Based on the present study, we propose potential explanations for this controversy. We accomplished this by measuring salivary oxytocin concentrations before and after conducting trust and risk games.

Our hypothesis was that oxytocin sensitivity varies greatly across individuals, and that performing subgroup analyses on oxytocin-sensitive participants would provide evidence for a causal link between oxytocin and trust. Our experiment revealed that oxytocin-sensitive participants receiving oxytocin tend to transfer more money to co-participants than do participants receiving a placebo. We also confirmed that oxytocin does not change attitude toward risk. These results indicate that oxytocin increases trust among humans, which is consistent with the results from Kosfeld et al. (2005).

Our trust and risk experiments were essentially the same as those of Kosfeld et al. (2005), to enable us to confirm the causal link between oxytocin and trust that those authors identified. Although Kosfeld et al. (2005) did not measure oxytocin, we confirmed the exact concentrations after oxytocin administration in the unextracted saliva, as validated by MacLean et al. (2018). Changes in concentrations before intranasal administration of oxytocin to after completion of the risk game seemed a reliable indicator, as evidenced by the linearity between measured values and exogenously added oxytocin concentrations in spiked samples. This was done to test our hypothesis that there is substantial variation in sensitivity to oxytocin between individuals, as well as to investigate the relationship of trust behavior with the initial oxytocin concentration and the administration-induced increase in the oxytocin concentration. In addition, we collected the participant’s saliva immediately before the trust game (but after oxytocin administration and interaction with other participants) in the final session of our experiment (*n* = 12), allowing us to verify whether the increase in oxytocin concentration was due to intranasal oxytocin administration or the interaction between investors and trustees in our trust game. Another difference between our studies was that the same participants participated in both the trust and risk games in our experiment, while Kosfeld et al. (2005) recruited different participants for the two games.

Our results are, however, weak compared to those of Kosfeld et al. (2005). There are two possible interpretations of the weak results: First, there are no effects of oxytocin on trust in humans; second, at minimum, the existing framework of the trust game makes it challenging to elicit the effect of oxytocin on trust in humans, or even trust itself. In fact, we found no significant difference in the mean average transfers between the oxytocin and placebo groups. Even when we considered sensitivity to oxytocin, we did not detect significant difference in the mean average transfers between groups. These results are consistent with the results of many studies reporting that replication attempts were not successful (Baumgartner et al., 2008; Barraza, 2010; Mikolajczak et al., 2010; Ebert et al., 2013; Klackl et al., 2013; Yao et al., 2014). A novel framework for eliciting trust in humans is needed, even though the effect of oxytocin on trust really exists.

The other interpretation is that the effects of oxytocin on trust in humans is person-dependent. Although we did not observe a significant difference in the mean average transfers between oxytocin and placebo groups, Fisher’s exact test revealed a statistically significant difference between oxytocin-sensitive participants and those in placebo groups in the proportions of investors who transferred the maximum amount of money. Measurement of salivary oxytocin concentrations revealed that this might have been due to large interpersonal variation in sensitivity to oxytocin. This corresponded to results from a study by Bendix et al. (2015), where plasma oxytocin levels varied across participants and was correlated to personality traits such as monotony avoidance and impulsiveness. In addition, previous studies indicate that the effect of oxytocin depends on personal conditions such as borderline personality disorder and attachment during childhood (Bartz et al., 2010b, 2011b). The positive effects on sociality also depend on receptor genotype (Marsh et al., 2012; Kosaka et al., 2016). In our study, performing subgroup analyses on oxytocin-sensitive participants improved the evidence for a causal link between oxytocin and trusting behavior. Oxytocin-sensitive participants tended to transfer more money upon oxytocin administration than upon placebo administration, while no such tendency was apparent for oxytocin-insensitive participants. These results indicate that the salivary concentration of oxytocin should be measured to allow researchers to control for such variation, and care is needed when interpreting the results of previous studies in the field. Specifically, previous evidence against a causal oxytocin-trust link might have been caused by inadvertent enrolment of a large proportion of oxytocin-insensitive participants. Measuring salivary oxytocin concentrations also enabled us to confirm whether increases in oxytocin concentration were primarily due to intranasal administration of oxytocin or interaction between participants during the trust game; our results were in favor of the former. In this study, we administered oxytocin via an intranasal route. When oxytocin diffuses into the brain and binds to oxytocin receptors, the neurons release oxytocin (Ludwig and Leng, 2006). Recently, MRI was used to monitor blood in the human brain, providing evidence that intranasally administered oxytocin reaches various brain regions (Martins et al., 2020). Those authors also reported that nasal oxytocin spreads via systematic circulation to the amygdala (Martins et al., 2020). In addition, recently, a molecule responsible for transporting oxytocin into the brain was identified: the receptor for advanced glycation end products (Yamamoto et al., 2019; Yamamoto and Higashida, 2020). These reports lend validity to our selected route of oxytocin administration.

In our experiment, the trust game started approximately 60 min after the administration of oxytocin and the risk game ended approximately 72 min after its administration. The task timing of the trust game is supported by Spengler et al. (2017), who reported that the oxytocin-induced inhibition of amygdala responses to fear was most effective in a time window between 45 and 70 min after administration of an oxytocin dose of 24 IU. As reviewed by Mierop et al. (2020), however, adequate time windows following intranasal oxytocin administration are unclear. Further research is needed to advance our understanding of the task timing.

In addition, we conducted a survey of participants’ AQ scores to analyze the effect of oxytocin on trust behavior in relation to autistic traits. Several researchers have revealed that oxytocin-induced prosocial behavior is correlated with indices used as proxies for autism, such as the EQ, AQ, and SQ indices. For instance, Hirosawa et al. (2012) discovered that oxytocin-induced prosocial behavior was correlated with lower EQ and higher SQ scores. On the other hand, in the large-scale study conducted by Yamasue et al. (2020), such a correlation was not clear for participants with autism. In this study, we observed that, in the oxytocin group, participants with lower AQ scores exhibited a statistically significantly higher degree of trust than did those with higher AQ scores. This is consistent with the results of Yamasue et al. (2020), in the sense that the effect of oxytocin was weak for participants with autistic traits. In contrast, Bartz et al. (2019), in a replication study of their previous research (Bartz et al., 2010a), discovered that oxytocin selectively improved empathic accuracy for men who scored higher on the AQ. One possible reason for the inconsistency in results between their study and ours is that there may be different mechanisms underlying empathic accuracy and trust among humans. However, care is needed when interpreting this result, as it may reflect individual variation in sensitivity to oxytocin. Even if recruitment is randomized as much as possible, a sample can still be biased toward oxytocin-sensitive or oxytocin-insensitive participants. Restricting the sample, for instance, to participants with a lower AQ score and a large increase in the level of oxytocin upon intranasal administration may produce clearer results. Unfortunately, the sample size of participants with autistic traits in this study was too small to perform such an experiment.

Our results should be interpreted with caution. Mierop et al. (2020) reported that the minimal sample size needed to detect an effect size of 0.28 is 404, as estimated by Walum et al. (2016), in a between-subjects design with a statistical power of 0.8 and a two-tailed type I error of 0.05. Declerck et al. (2020) reported that a sample size of 166 is needed to detect an effect size of 0.514, the reported effect size in Kosfeld et al. (2005), with a statistical power of 0.95 and a one-tailed type I error of 0.05. The effect size of our main analysis (0.28) was smaller than the minimal detectable effect size with a statistical power of 0.8 and a one-tailed type I error of 0.05 (0.58). Upon performing a two-sided *t*-test with the null hypothesis that our effect size was equal to the minimal detectable effect size, we could not reject the null hypothesis [*t*_(__94__)_ = 1.39, *P* = 0.16]; this indicated that we could not conclude that our effect size was larger than the minimal detectable effect size. The effect sizes of our subsample analyses for oxytocin sensitivity (*d* = 0.34) and the AQ scores (*d* = 0.40) were also not significantly different from the minimal detectable sizes [minimal detectable sizes = 0.63 and 0.64, respectively; *t*_(__82__)_ = 1.15 and *t*_(__77__)_ = 1.01, respectively; *P* = 0.26 and 0.31, respectively], a clear limitation of our results. In addition, our reported effects had relatively large *p*-values using one-sided tests. Moreover, the statistical significance of the differences in effect sizes did not persist after Bonferroni corrections. These limitations of our study indicate the importance of conducting studies with sufficient sample size to detect an effect. Although this kind of research is resource intensive and costly, which makes it difficult to obtain large samples (Bartz et al., 2019), better-powered studies with larger sample sizes are needed to further our understanding in the field. Furthermore, as Mierop et al. (2020) suggested, transparent research practices such as preregistration of studies stating relevant theories, hypotheses, measures, analyses, and power estimations are needed to obtain a solid empirical basis for the field. Our study, however, was not a preregistered study, which lacks transparency. This is also a limitation of our study. In conclusion, our results point to a correlation between oxytocin administration and trust (in the form of money transfer to trustees in a trust game), although there were many limitations that may have undermined the repeatability of these findings.

## Data Availability Statement

The datasets presented in this study can be found in online repositories. The names of the repository/repositories and accession number(s) can be found below: https://github.com/YusukeKinari/Kurokawa-et-al.-2021-.

## Ethics Statement

The studies involving human participants were reviewed and approved by the Osaka University Ethics Committee. The patients/participants provided their written informed consent to participate in this study.

## Author Contributions

HK coded the program used for our experiment and processed the data. KT, YS, MK, and HH were responsible for acquisition of the biological data and measurement of the salivary oxytocin concentrations. HK, YK, HO, and FO were responsible for acquisition of the behavioral data and analyzed the behavioral and biological data. YK interpreted the results and wrote the draft. All authors read, edited, approved the final manuscript, and contributed to the study design.

## Conflict of Interest

The authors declare that the research was conducted in the absence of any commercial or financial relationships that could be construed as a potential conflict of interest.
