# Supplementary material for: Oxytocin-Trust Link in Oxytocin-Sensitive Participants and Those Without Autistic Traits
Source: Front Neurosci. 2021 May 25;15:659737. doi: 10.3389/fnins.2021.659737 (PMC8186783; doi:10.3389/fnins.2021.659737)

鼻用定量噴霧器の使用方法

①鼻をかんで鼻腔の通りをよくしてください。

②キャップをはずし、矢印の方向によく振ってください。

③新しいスプレーで最初の吸入を行う時は、必ず予備噴霧として 2 噴霧してください。容器の底を親指で支え、人差し指と中指でノズルの両端を持ちます。垂直の状態で親指だけを動かす様にしてノズルが確実に止まるところまで一気に押し上げて噴霧します。2 噴霧しても薬液が出ない場合は押す力が弱いことが考えられますので、もう少し強く一気に押してください。

④頭をうつむき加減にして、片方の鼻孔をふさぎ他方の鼻孔内に噴霧器の先端を垂直に立てて入れてください。人差し指と中指が鼻の下端に軽く触れる状態にして、この 2 本の指の位置を固定します。鼻から息を吸い込みながら、ノズルが確実に止まるところまで一気に押し上げて噴霧・吸入します。

⑤噴霧後は一回ずつ、薬剤を鼻の奥まで行き渡らせるために、頭を後に傾けた状態で数秒間鼻から静かに呼吸してください。左右の鼻孔に交互に 3 回ずつ噴霧します。つまり、左→右→左→右→左→右と噴霧します。

⑥使用後は噴霧器の先端をきれいにふき、必ずキャップをしてください。次回使用するまでは、冷蔵庫で保管しておいてください。

＜1 本あたり、4 日分の使用量に相当しています＞

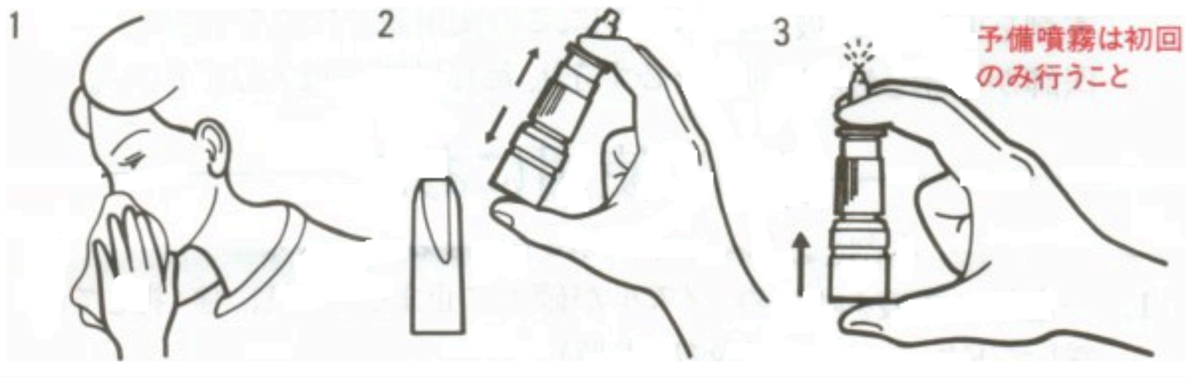

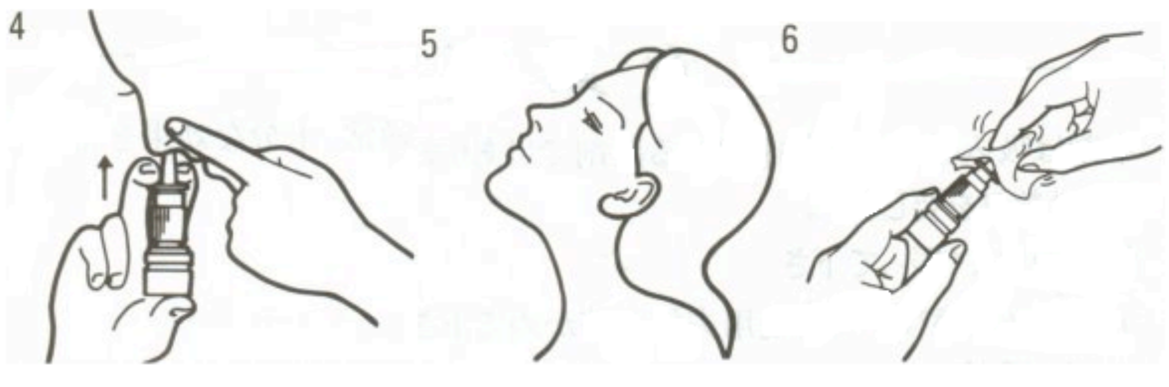

Supplement: Supplementary file 1 [file Data_Sheet_1.PDF]
